# Tailoring a Refractory High Entropy Alloy by Powder Metallurgy Process Optimization

**DOI:** 10.3390/ma14195796

**Published:** 2021-10-03

**Authors:** Larissa Moravcikova-Gouvea, Igor Moravcik, Vaclav Pouchly, Zuzana Kovacova, Michael Kitzmantel, Erich Neubauer, Ivo Dlouhy

**Affiliations:** 1 Institute of Materials Science and Engineering, Brno University of Technology, Technicka 2896/2, 61669 Brno, Czech Republic; igor.moravcik@vutbr.cz (I.M.); dlouhy@fme.vutbr.cz (I.D.); 2 Central European Institute of Technology (CEITEC), Purkynova 123, 61200 Brno, Czech Republic; pouchly@fme.vutbr.cz; 3 RHP-Technology GmbH, Forschungs- und Technologiezentrum, 2444 Seibersdorf, Austria; z.ko@rhp.at (Z.K.); m.ki@rhp.at (M.K.); e.ne@rhp.at (E.N.)

**Keywords:** refractory complex concentrated alloys, microstructures, mechanical alloying, spark plasma sintering, mechanical properties

## Abstract

This paper reports the microstructural evolution and mechanical properties of a low-density Al_0.3_NbTa_0.8_Ti_1.5_V_0.2_Zr refractory high-entropy alloy (RHEA) prepared by means of a combination of mechanical alloying and spark plasma sintering (SPS). Prior to sintering, the morphology, chemical homogeneity and crystal structures of the powders were thoroughly investigated by varying the milling times to find optimal conditions for densification. The sintered bulk RHEAs were produced with diverse feedstock powder conditions. The microstructural development of the materials was analyzed in terms of phase composition and constitution, chemical homogeneity, and crystallographic properties. Hardness and elastic constants also were measured. The calculation of phase diagrams (CALPHAD) was performed to predict the phase changes in the alloy, and the results were compared with the experiments. Milling time seems to play a significant role in the contamination level of the sintered materials. Even though a protective atmosphere was used in the entire manufacturing process, carbide formation was detected in the sintered bulks as early as after 3 h of powder milling. Oxides were observed after 30 h due to wear of the high-carbon steel milling media and SPS consolidation. Ten hours of milling seems sufficient for achieving an optimal equilibrium between microstructural homogeneity and refinement, high hardness and minimal contamination.

## 1. Introduction

The requirements for designing novel metallic alloys that are capable of surpassing a whole set of specific properties of currently available commercial alloys are, at present, a subject of high demand in the industry. For instance, an improved combination of metallic alloys should possess strength and ductility while maintaining a relatively low cost. This is especially true when one is dealing with materials for high-temperature applications. Therefore, new engineering solutions are needed for novel materials that outperform the capabilities of the currently used Ni-based alloys. One of the prospective innovative materials that could be able to meet the requirements of such demanding applications, especially in the aviation and aerospace industries, may be refractory high-entropy alloys (RHEAs). These are also known by other names, such as complex concentrated alloys (CCAs). These materials have been extensively studied in recent years [1,2,3,4,5,6,7] due to their very interesting combination of properties in some of the compositions.

Yet, RHEAs are also able to exhibit superalloy-like microstructures [2,8,9,10], often possessing coherent BCC_A2 and BCC_B2 phase relationships similar to those FCC_A1 and FCC_L1_2_ structures exhibited by Ni-based superalloys [11]. These are responsible for the excellent high-temperature stability and properties of these alloys. The main challenge in dealing with refractory alloys is that they are often brittle [12,13,14], especially at room temperature. However, under certain processing conditions, these novel RHEAs possess a high strength combined with a satisfactory ductility level at both room and high temperatures [4,14,15,16], performing superiorly compared to some of the commercial Ni-based superalloys [1]. In particular, the Al_0.5_NbTa_0.8_Ti_1.5_V_0.2_Zr composition and its variants have stood out as potential alternatives for the production of strong and ductile refractory high-entropy superalloys [15,16,17].

HEAs are widely produced by liquid state methods, such as casting by vacuum arc-melting, induction melting, etc. [18,19]. However, for refractory alloys, these production routes may become challenging and costly, as these alloys possess very high melting temperatures. Thus, segregation of certain high-melting temperature elements may occur, as for the evaporation of low melting ones.

The powder metallurgy (PM) method offers a wide range of possibilities for the fabrication of alloys in the solid state [12]. For refractory alloys, liquid state processing may become challenging and costly, as these alloys possess very high melting temperatures. Therefore, using mechanical alloying and subsequent sintering as a feasible production route of RHEAs may provide several advantages in the material, such as the avoidance of segregation effects and the production of homogeneous materials with small average grain sizes, thus leading to the production of high-strength materials [20,21]. Due to the better homogeneity as compared to liquid routes, lack of preferential texture is often a characteristic of these materials due to the nature of the process [22], lacking any cold deformation after sintering. This method can be scaled up by the simple use of larger identical equipment and applied in industry, without any other alterations. On the other hand, the method is highly susceptible to contamination, and therefore the formation of oxides/carbides is nearly unavoidable, even when one uses controlled atmospheres [22,23,24]. However, one may exploit the formation of oxides/carbides as a way of exploring the oxide dispersed strengthening (ODS) effect [12,25,26,27], which may play an important role in the strengthening mechanisms of HEAs [28,29].

In this light, the present work focuses on understanding the effects of the variation of powder metallurgy parameters on the microstructure and mechanical properties of Al_0.3_NbTa_0.8_Ti_1.5_V_0.2_Zr RHEA powders and the respective produced bulks by manufacturing the materials by mechanical alloying and sintering. The variation in the milling time on the powders may greatly affect the final materials. The RHEA microstructures and properties were thoroughly investigated by means of X-ray diffraction (XRD), scanning electron microscopy (SEM) combined with energy dispersive X-ray spectroscopy (EDS) and electron backscattered diffraction (EBSD) technique, micro- and nanoindentation hardness, and ultrasound methods to assess the elastic properties of the materials. Calculation of phase diagrams (CALPHAD) were assessed to predict the phase changes associated with the process, and the results were compared with the experiments.

## 2. Materials and Methods

### 2.1. Mechanical Alloying and Sintering

The Al_0.3_NbTa_0.8_Ti_1.5_V_0.2_Zr alloy (stoichiometric ratio) was prepared by means of a combination of mechanical alloying (MA) and spark plasma sintering (SPS). The following elemental powders with commercial purity were used as feedstock: Al (99.9 wt.% purity, Sigma Aldrich, USA), Ti, Nb, V (over 99.5 wt.% purity, Alfa Aesar, Karlsruhe, Germany), Ta (99.9 wt.% purity, CRM, China) and Zr powder (99.5 wt.% purity, TLS Technik, Bitterfeld-Wolfen, Germany). The powders were handled before MA under argon in a high-purity atmosphere in a glovebox.

The precursor powders were mechanically alloyed in a Fritsch Pulverisette 6 planetary ball mill under different milling times to evaluate the evolution of their morphology and microstructure with the time of milling. The selected milling times were 0 (these were not milled, rather simply mixed together in a horizontal blender for 10 min for homogeneous blending), 1, 3, 5, 10, 20, 30, 40, and 50 h. The milling was carried out under a high-purity argon atmosphere (99.9999%) in a sealed bearing steel bowl containing hardened steel ball bearings (AISI 52100) of 15 mm in diameter in a 10:1 ball-to-powder weight ratio (BPR). The milling was conducted with a milling speed of 250 RPM in the system—the milling jar and the platform bottom were connected by an arm gauge and span in synchrony, as shown in other works [30]. One cycle consisted of 15 min of milling and 1 h idle time until the maximum milling time was reached, e.g., 1 h of milling corresponded to 4 cycles (which comprised 1 h of active milling + 4 h of idle time). This cycling scheme was chosen to avoid overheating of the system, and therefore to avoid any formation of potential oxides caused by the overheating of the milling jar. Wet milling in toluene for an additional 20 min was necessary to remove the powder stuck to the milling balls’ surfaces.

The sintering was performed on selected powders subject to different milling times, namely 3, 10, 30 and 50 h. This was performed to evaluate the effect of the milling time on the final properties of the sintered bulks. The milled powders were consolidated by spark plasma sintering technology (Sumitomo Coal Mining, Dr. Sinter SPS machine) using a graphite die with an inner diameter of 12 mm in a vacuum atmosphere. The sintering parameters were selected according to a previous methodology optimization [12] performed in RHP-Technology GmbH, Austria. The sintering was carried out at a constant uniaxial pressure of 50 MPa applied by die pistons at a maximum temperature of 1200 °C and a heating rate of 100 °C·min^−1^ up to 50 °C from the maximum sintering temperatures. For the last 50 °C, a heating rate of 25 °C·min^−1^ was used with a dwell time of 15 min. After sintering, the machine was turned off and the samples naturally cooled down in vacuum.

### 2.2. Characterization

The samples studied in this work for the microstructural observations were prepared by hot mounting in conductive resin and mechanical grinding with SiC abrasive papers down to #2400 grit. The samples were polished using 3 and 1 μm diamond paste. The last steps of the sample preparation were mechano-chemical polishing using a Struers OPS colloidal silica suspension and vibration polishing for a minimum of 4 h using an Eposil M colloidal silica suspension of 0.06 μm.

Scanning electron microscopy (SEM) using a ZEISS Ultra Plus FEG microscope was used in all materials for the analysis of the microstructural features, using secondary electron (SE) and backscattered electron (BSE) detectors. For the chemical composition, the energy-dispersive X-ray spectroscopy (EDS) mode was used. The compositional values are an average of at least three measurements, where the error is the standard deviation. Electron backscatter diffraction (EBSD) was utilized for obtaining information about the structure, crystal orientation, phase, grain size average and other structural parameters.

X-ray diffraction (XRD) analysis for detailed structural characterization, phases, chemical composition and geometrical parameters was performed in all materials. This was performed mainly using a Philips X’Pert Pro diffractometer operating at 40 kV voltage with a current of 30 mA. Continuous scanning was usually performed with 2θ between 30° and 100° using a speed of 0.02°·min^−1^ and a step size of 0.0167°. The radiation used was Cu-Kα with λ = 1.54056 Å.

Vickers hardness was assessed for all materials using microhardness testing according to ISO 6507-1:2005 and using a LECO LM 274 AT hardness tester, with applied loads of 0.2 kgf (1.961N force) at 10 s of dwell time. The individual results for materials represent an average of at least 15 measurements, and the error is the standard deviation.

Nanoindentation testing was carried out in all materials to calculate hardness and elastic modulus of the materials, according to the Oliver and Pharr method [31], which uses the unloading curve for the elastic modulus calculation [32,33,34]. The CSM Instruments NHT2 nanoindentation tester equipped with a Berkovich diamond indenter was used at an acquisition rate of 10 Hz, with a maximum load of 100 mN, loading and unloading rates of 200 mN·min^−1^, and a dwell time of 10 s. Averages were taken from at least 15 indents for each sample, and the error is the standard deviation of the values.

Ultrasound (US) measurements were carried out to investigate the influence of the sintering times on its final properties. The tests were performed using an Olympus 38DL Plus system for elastic modulus and Poisson ratio calculations. The probe used for the determination of elastic modulus was the M112 10MHz Longitudinal wave transducer and the probe used for obtaining the Poisson ratio and shear moduli was the V156 5 MHz Shear wave transducer.

Calculation of phase and property diagrams was carried out using ThermoCalc software version 2020b (TCHEA4.1 database) for prediction of equilibrium phases.

## 3. Results

### 3.1. Phase Prediction

**Figure 1:**
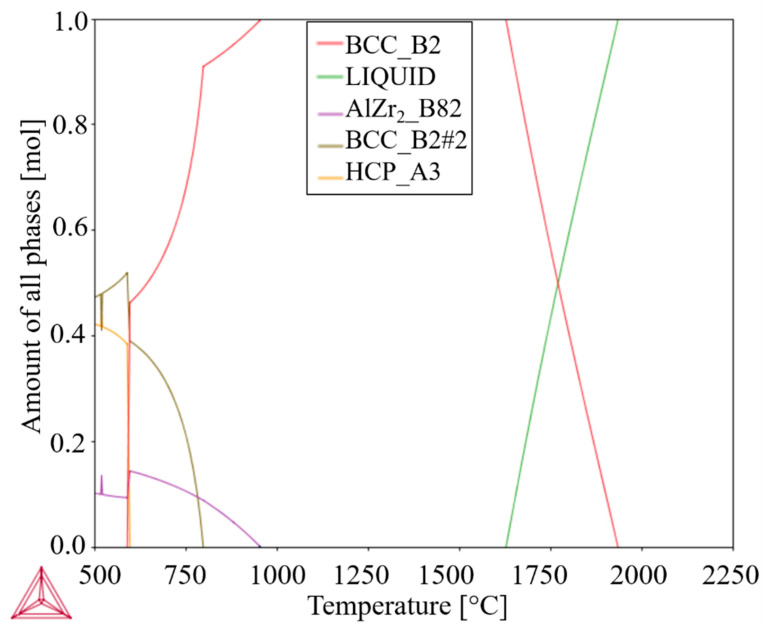
Computed phase diagram for Al_0.3_NbTa_0.8_Ti_1.5_V_0.2_Zr RHEA according to CALPHAD calculations using ThermoCalc software, as the amount of all phases vs. temperature (for interpretation of the references to color in this figure legend, the reader is referred to the web version of this article).

In order to evaluate the possible phases present in the Al_0.3_NbTa_0.8_Ti_1.5_V_0.2_Zr RHEA, the equilibrium property diagram of the selected alloy was computed using the ThermoCalc software. The calculated diagram is exhibited in Figure 1.

According to the calculated equilibrium property diagram, the melting temperature of the Al_0.3_NbTa_0.8_Ti_1.5_V_0.2_Zr alloy is approximately 1930 °C. Below this temperature down to 1630 °C, nucleation of a BCC_B2 should take place until it reaches a single-phase BCC_B2 microstructure. For temperatures under approximately 950 °C, nucleation of the AlZr_2__B8_2_ phase should occur. At 800 °C, it is predicted that a concurrent nucleation and growth of a second BCC_B2 phase takes place. Further temperature reduction below 600 °C induces the formation of HCP_A3.

To produce the alloy in this study, the sintering temperature of 1200 °C was used. At this temperature, equilibrium CALPHAD calculations indicate that the only phase in the Al_0.3_NbTa_0.8_Ti_1.5_V_0.2_Zr alloy should be BCC_B2.

### 3.2. Microstructural and Morphological Characterization of the Powders

**Figure 2:**
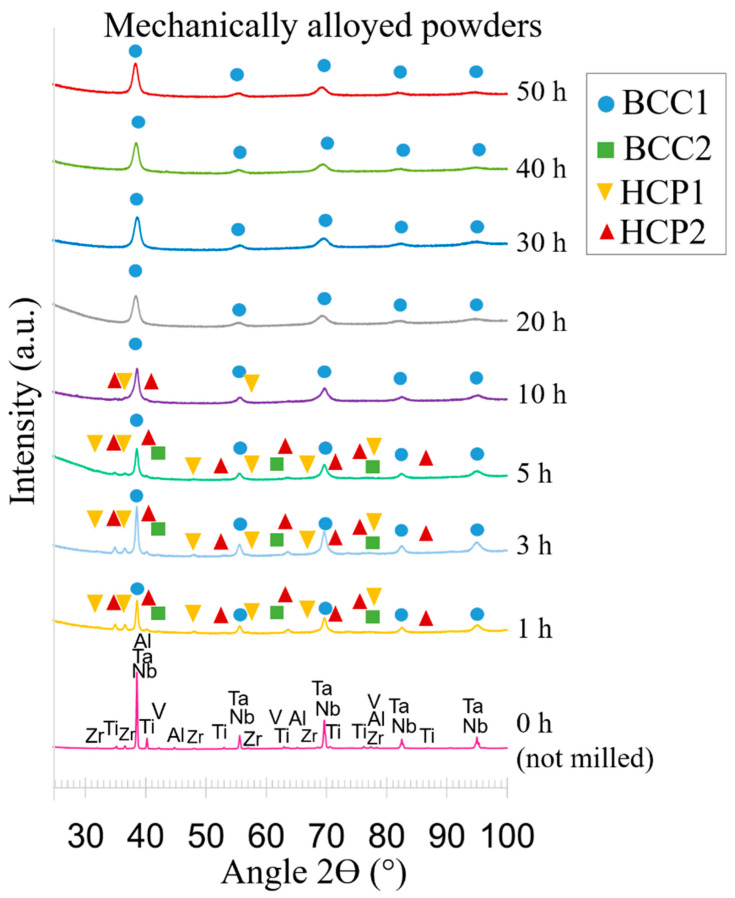
XRD patterns of Al_0.3_NbTa_0.8_Ti_1.5_V_0.2_Zr powders after various milling times.

The RHEA Al_0.3_NbTa_0.8_Ti_1.5_V_0.2_Zr (in stoichiometric ratio) was prepared by mechanical alloying followed by spark plasma sintering. The mechanical alloying was performed using a variety of milling times—while keeping the same milling setup conditions—to evaluate the influence of the milling time on the powders and bulks properties. The milling was carried out for different milling times and the non-milled powders are referred to as 0 h, which were investigated for comparison. The XRD patterns of the mechanically alloyed powders for the different times can be seen in Figure 2, while the respective lattice parameters calculated from the XRD patterns are shown in Table 1.

The unmilled powder (0 h) shows peaks pertaining to elemental feedstock pure powders. These are: Al with a = 4.05 Å, space group Fm-3m, in 1.9 wt.%; Nb with a = 3.30 Å, space group Im-3m, in 22.2 wt.%; Ta with a = 3.31 Å, space group Im-3m, in 34.6 wt.%; Ti with a = 2.95 Å, c = 4.68 Å, space group P63/mmc, in 17.1 wt.%; V with a = 3.03 Å, space group Im-3m, in 2.4 wt.%; Zr owning a = 3.23 Å, c = 5.15 Å, space group P63/mmc, in 21.8 wt.%. This is in good agreement with the values in wt.% for this alloy, which correspond to Al_1.93_Nb_22.17_Ta_34.55_Ti_17.14_V_2.43_Zr_21.77_. This shows that the powders in the initial condition had a precise composition as calculated by the CALPHAD phase diagram. The same alloy in at.% has an Al_6.25_Nb_20.80_Ta_16.68_Ti_31.25_V_4.17_Zr_20.80_ stoichiometry.

Solid solution forming reactions should occur upon the start of the milling process of the mixed powders [35]. Therefore, after 1 h of mechanical milling, the powder exhibits four different phases, which are denominated here as: BCC1, BCC2, HCP1 and HCP2. Note that these phases after only 1 h of milling are probably compositionally very close to the pure elements from which they originate. The major phase is BCC1, detected by the COD ID 90088552, which is related to the peaks of Nb and Ta before mechanical alloying. After milling, these peaks completely overlap due to the very similar crystal structures of both elements and probably the dissolution of these elements in the lattices of one another, to some extent, caused by mechanical alloying.

The second richest phase is HCP1, belonging to the P63/mmc space group (COD ID 9008523). These peaks are related to those of the pure non-milled Zr, which might, after milling, contain elements that were mechanically alloyed to the crystal lattice, changing the nature of the original phases. This is due to deformation and a slightly altered chemistry compared to the pure powder. Note that even the wt.% of the HCP1 is different to that of pure Zr.

The HCP2 structure belongs to the P63/mmc space group too (COD ID 9008517), structure type Ti. The Al element was successfully mechanically alloyed to the other powders in its total content already after 1 h. This is due to the absence of the peaks of this element.

After 3 h of milling, BCC1, HCP2 and BCC2 are present. After 5 h of milling, BCC1 significantly increased its content. Concurrently, the remaining phases substantially decreased. After 10 h of milling, the BCC1 is clearly dominant, reaching almost the whole weight of the alloy. The BCC2 is completely dissolved in the lattices of the other phases. HCP1 is present.

After 20 h of milling, a single BCC1 solid solution is already achieved. Subsequent milling for 30, 40, and 50 h does not induce any phase changes.

**Figure 3:**
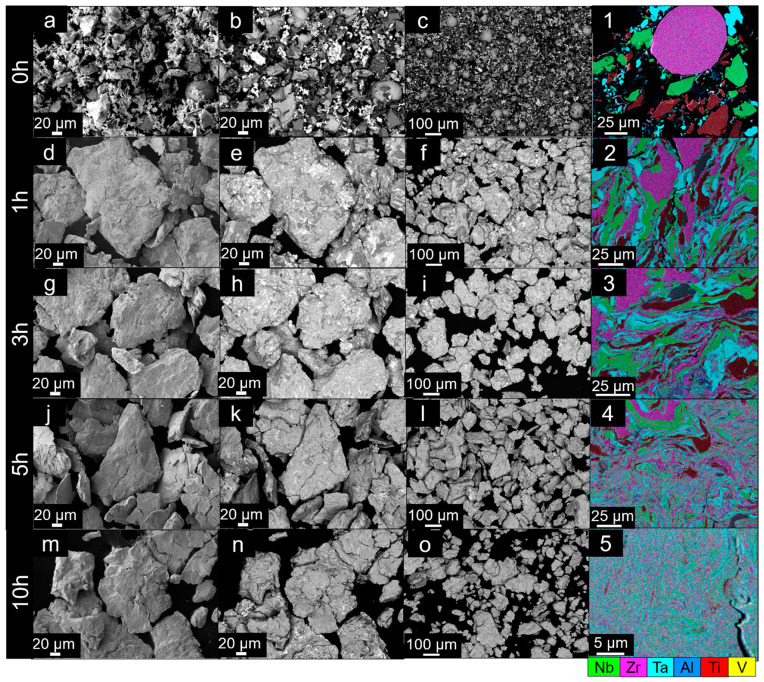
SEM images of Al_0.3_NbTa_0.8_Ti_1.5_V_0.2_Zr powders after different milling times from 0 to 10 h, respectively. The milling times are noted on the left side of each row of images. (**a**,**d**,**g**,**j**,**m**) SEM micrographs using SE detector showing powders in detail after each milling from 0 to 10 h, in order. (**b**,**e**,**h**,**k**,**n**) Corresponding SEM images using a BSE detector showing powders in detail after each milling. (**c**,**f**,**i**,**l**,**o**) SEM images using BSE mode to exhibit an overview of the powders in lower magnifications. (**1**–**5**) EDS maps with all elements in color are shown, taken from the particle cross-section of each powder milled from 0 to 10 h, respectively.

**Figure 4:**
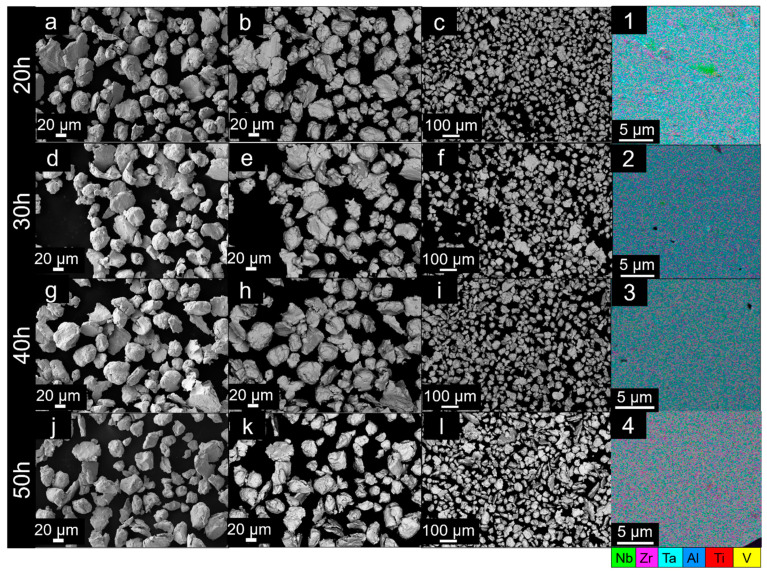
SEM images of Al_0.3_NbTa_0.8_Ti_1.5_V_0.2_Zr powders after different milling times from 20 to 50 h, respectively. The milling times are shown on the left side of each row of images. (**a**,**d**,**g**,**j**) SEM micrographs using SE detector showing powders in detail after each milling from 20 to 50 h, in order. (**b**,**e**,**h**,**k**) Corresponding SEM images using BSE detector showing powders in detail after each milling. (**c**,**f**,**i**,**l**) SEM images using BSE mode to exhibit an overview of the powders in lower magnifications. (**1**–**4**) EDS maps taken from the particle cross-section of each powder milled from 20 to 50 h, respectively.

The SEM analyses of the milled powders are shown in Figure 3 for milling times from 0 to 10 h and Figure 4 for 20 to 50 h. The respective EDS maps taken from the cross-section of each milled powder are shown in the last column of the set of micrographs.

Prior to mechanical alloying, the elemental powders exhibited diverse morphologies and sizes, as visible in Figure 3a–c,1. However, after only 1 h of milling, the powders start to acquire similar particle sizes, and are effectively mechanically alloyed, with significant deformation and alloying of all elements. This is visible in the EDS maps of the cross-sections of these 1 h milled powders (Figure 3(2)).

Further milling for 3 and 5 h brings about significant alloying of all elements and chemical homogenization. However, it is possible to still observe the segregation of elements, especially Zr and Ti, which possess HCP crystal structures.

After 10 h of milling, one can observe that the powders start to have internal cracks (Figure 3(5)) and fracture into smaller particles (Figure 3o). Concurrently, the powders are virtually fully homogeneously alloyed, and no significant segregation can be noticed (Figure 3(5)).

From 20 h milling, one can expect again a more homogeneous distribution of particle sizes in a globular morphology (Figure 4a–c,1), with a significant decrease in size as compared to the 10 h state.

The microstructures for milling times of 30 and 40 h do not show significant variations in powder morphology and sizes, but the homogenization of the fully mechanically alloyed powders is achieved.

After 50 h of milling, it is possible to see that some particles are elongated (Figure 4(l)), but the powders are mostly of a globular morphology.

### 3.3. Microstructural Characterization of Refractory High-Entropy Alloy Bulks

The RHEA Al_0.3_NbTa_0.8_Ti_1.5_V_0.2_Zr bulks were prepared from powder previously milled at varying times. To characterize their microstructures and mechanical properties, and to additionally analyze the effect of the milling time variation on the bulks. Note that identical conditions of sintering, such as heating rates, vacuum level, dwell times at the maximum temperature, and cooling rates were kept constant.

**Figure 5:**
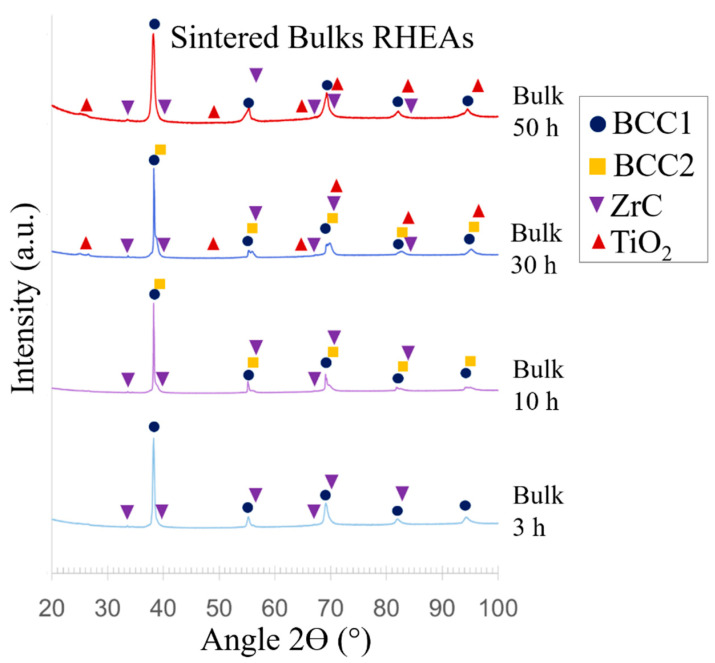
XRD patterns of sintered bulks using powders subject to different times of milling.

The selected milled powders were 3, 10, 30 and 50 h. XRD patterns of the bulks are shown in Figure 5.

The sintered bulk using feedstock powders milled for 3 h shows a main BCC1 phase and a small fraction of carbides, belonging to the crystal structure of ZrC, space group Fm-3m (ICSD Coll. Code 26954). On the other hand, when the bulks are prepared from 10 h milled powders, they exhibit two BCC phases, instead of one. These are called BCC1 and BCC2. This is evidenced by the additional peaks of BCC2 in very close angles from those of BCC1—thus demonstrating very close lattice parameters to the ones of BCC1. The exact lattice constants of each phase are found in Table 2.

The bulk manufactured from the 30 h milled powder shows the BCC1 matrix; the ZrC fraction remains relatively unchanged. Additionally, the crystal structure of TiO_2_ appears, belonging to the tetragonal crystal system and I41/amd space group (COD ID 5000223). Finally, the bulk prepared from the 50 h milled powder shows the formation of a main BCC1, while BCC2 is interestingly absent. The remaining fraction corresponds to oxide formation. The ZrC fraction increases with increasing milling time.

**Figure 6:**
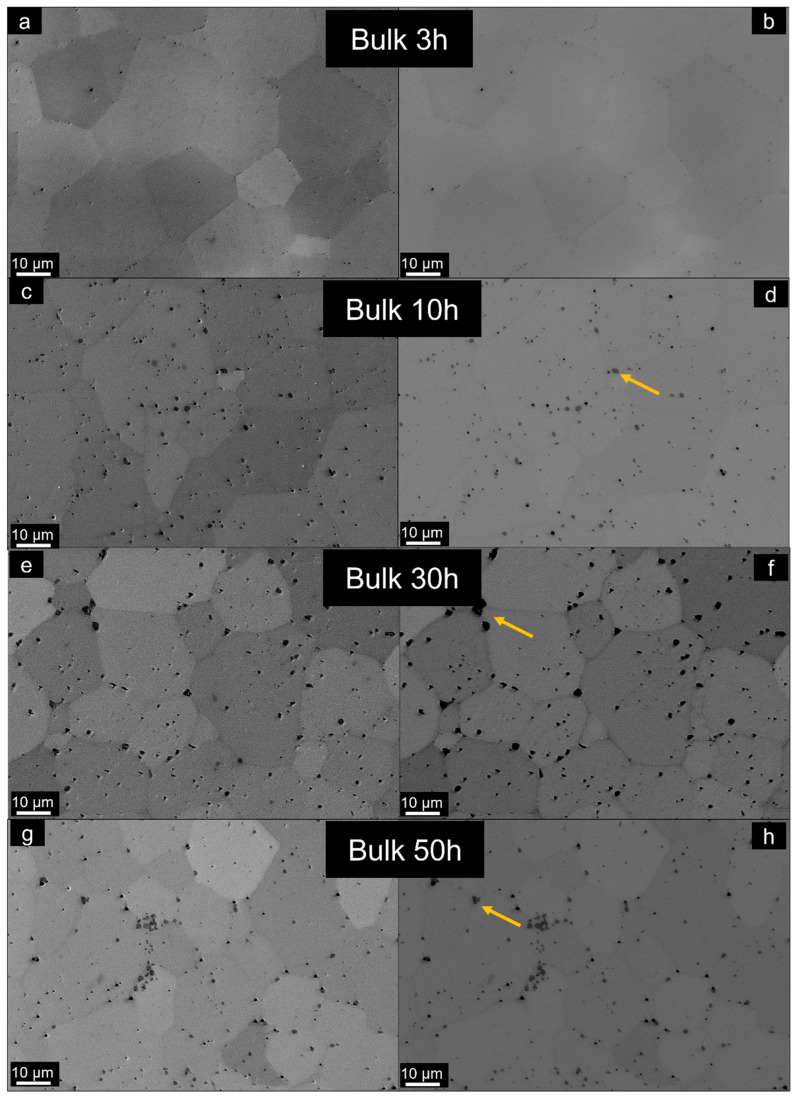
SEM micrographs of the Al_0.3_NbTa_0.8_Ti_1.5_V_0.2_Zr sintered bulks produced with different powder milling times in SE (left column) and BSE (right) modes, respectively: (**a**,**b**) 3 h, (**c**,**d**) 10 h, (**e**,**f**) 30 h, (**g**,**h**) and 50 h. The yellow arrows denote the oxide and carbide particles formed due to contamination.

**Figure 7:**
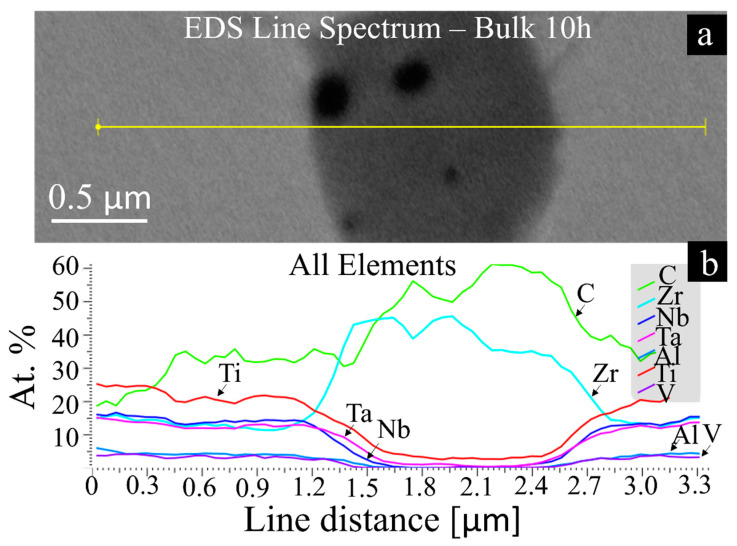
Representative EDS line spectrum performed on Bulk 10 h showing: (**a**) SEM image with the location of the line; (**b**) the chemical composition distribution of the dark particles present in all RHEAs in terms of atomic percentage vs. distance.

The microstructures of the sintered bulks are revealed in Figure 6. The micrographs reveal a BCC matrix with dispersed carbides in all materials, especially visible around the grain boundaries as dark particles which are marked with yellow arrows. An EDS line spectrum was performed on these dark particles and the results can be seen in Figure 7, which clearly shows that these areas belong to ZrC particles. The 3 h bulk RHEA shows fewer carbides than all other conditions, in agreement with the XRD results. Further milling significantly increases the carbides content, especially for 30 and 50 h, where two crystal structures composed of carbides and oxides are present (from cubic (ZrC) and tetragonal (TiO_2_) systems). EDS area analyses were performed in the BCC matrix, which are shown in Table 3.

It is evident that the BCC1 phase possesses similar compositions for all sintered bulks according to EDS. However, the presence of Fe at 1.8 at.% is manifested after 30 h of milling. The presence of Fe increases to 2.2 at.% for 50 h of milling time. Note that Fe was detected also in the materials milled for 3 and 10 h, but was not presented due to the uncertainty of the EDS measurement for such small concentrations. Fe’s presence is an indicator of powder and bulk contamination during milling due to the use of carbon steel milling media [24].

**Figure 8:**
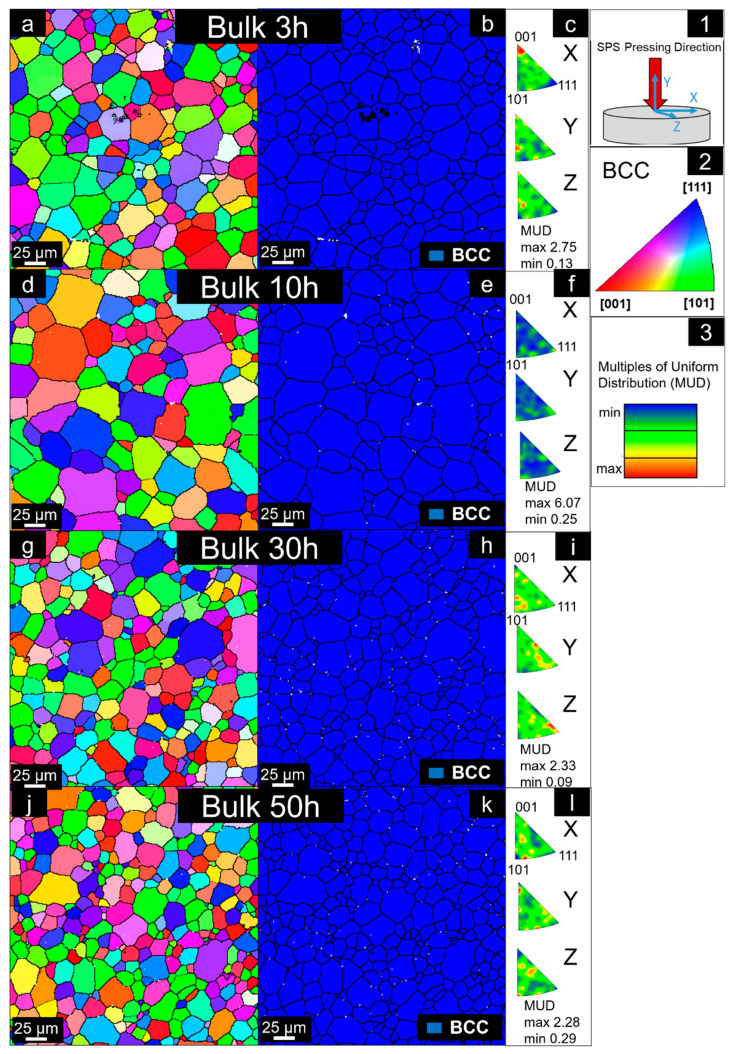
Representative EBSD results of Al_0.3_NbTa_0.8_Ti_1.5_V_0.2_Zr sintered bulks subject to different powder milling times. (**a**,**d**,**g**,**j**) Inverse pole figure showing grain orientation maps with reference direction perpendicular to the SPS compaction direction denoted as Z in (**1**), for 3, 10, 30 and 50 h, respectively. The colors represent the directions shown in (**2**). (**b**,**e**,**h**,**k**) Phase map showing distribution of BCC grains (white regions represent dispersed oxides), for 3, 10, 30 and 50 h, respectively. (**c**,**f**,**i**,**l**) Inverse pole figures of BCC phase. MUD reference is shown in (**3**).

For quantitative results, EBSD was performed in all samples and can be seen in Figure 8 along with their values of multiples of uniform distribution (MUD values).

The bulk from the 3 h milled feedstock powder shows a BCC matrix with an average grain size of 33.8 µm. The white regions represent the secondary ZrC phase shown in the XRD. Its calculated average grain size is 1.8 µm according to four EBSD measurements in different areas. The bulk made using the 10 h milled powder also exhibits a BCC matrix, with an average grain size of 37.7 µm and dispersed oxides in the white areas. The calculated average grain size of these regions is 3.3 µm. It is not possible to assess the second BCC phase shown in XRD due to the resolution limit of the EBSD. This phase probably has a nano-sized character, as previously exhibited by other authors in similar RHEA compositions, showing a BCC_A2/BCC_B2 relationship [16].

The bulks made by utilizing 30 and 50 h milled feedstock powders show more refined microstructures in comparison with the previous states. The average grain sizes of the BCC phase in both materials are calculated to be 28.3 and 25.2 µm, respectively. There is a weak preferential orientation in [001] in the X axis for the Bulk-3h, caused probably by a slight plastic flow during pressing, locally in this specific region of the EBSD map. However, it is not intense enough to be relevant or to be considered as a preferential texture (as its maximum MUD is too low, at the same level as that of the other samples). The random orientation of the grains, despite the uniaxial pressing during SPS, is clearly maintained in all samples.

### 3.4. Mechanical Properties

**Figure 9:**
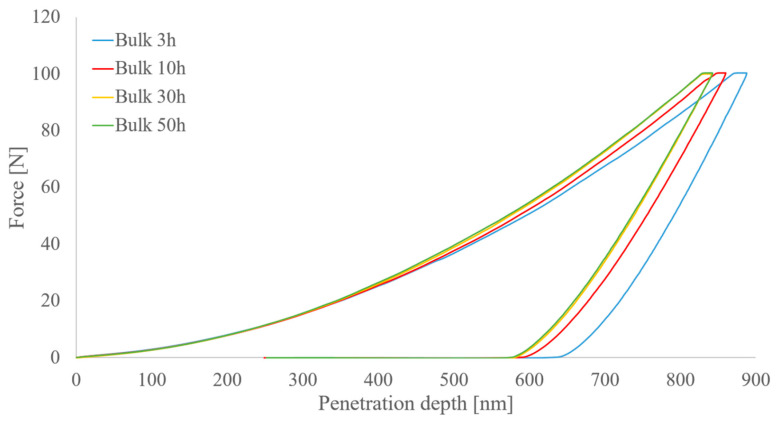
Representative plots of applied force vs. penetration depth from the nanoindentation hardness test of the Al_0.3_NbTa_0.8_Ti_1.5_V_0.2_Zr RHEA bulks. The bulk produced with mechanically alloyed powder for 3 h has the highest values of penetration depth in comparison with the others.

The mechanical properties of the Al_0.3_NbTa_0.8_Ti_1.5_V_0.2_Zr RHEA bulks were assessed by microindentation hardness tests, nanohardness tests, and ultrasound methods. Comparative results for all samples are presented in Table 4 and Figure 9.

The RHEA produced by mechanical alloying of powders for a period of 50 h presented the highest hardness of 675.7 ± 9.8 HV0.2 among all alloys, while the lowest hardness of 592.9 ± 21.0 HV0.2 was exhibited by the alloy produced with the minimum milling time of 3 h.

This trend also agrees with the penetration depth values obtained by the nanoindentation hardness tests. The highest penetration depth (893.9 ± 5.2 nm) was achieved by the alloy produced from 3 h milled feedstock powders in comparison with the lowest penetration depth of 842.6 ± 3.6 nm of the bulk produced with 50 h milled powders. The elastic modulus, as reflected by the Oliver–Pharr method and also by the slope of the unloading part of the nanoindentation curves, shows a slight increase with the increase in hardness, from 134.7 GPa up to 138.7 GPa.

A representative plot of force vs. penetration depth is exhibited in Figure 9, where the maximum penetration depth of the produced RHEA bulks is directly related to the time of milling of the feedstock powders, thus influencing the hardness of the materials. Under the same applied force of 100 mN, the lowest hardness is related to the highest penetration depths, and it is shown by the alloy produced by milling for 3 h. With increasing milling times, the hardness proportionally increases and the penetration depths decrease.

Additionally, for the Al_0.3_NbTa_0.8_Ti_1.5_V_0.2_Zr RHEA bulk produced with 10 h milled feedstock powders, the results of the elastic moduli by both nanoindentation hardness and ultrasound methods, along with the Poisson ratio and shear modulus values, are presented in Table 5. The elastic modulus from both methods shows similar results for this specific time of milling (≈136 GPa), while the Poisson ratio was calculated to be 0.326.

For reference, the hardness values of several other HEAs, including RHEAs, either prepared by powder metallurgy (PM) or arc-melting, are shown in Table 6.

## 4. Discussion

### 4.1. Influence of the Milling Time on the Properties of the Powders

The consequences of the variation in the milling time on the properties of the Al_0.3_NbTa_0.8_Ti_1.5_V_0.2_Zr RHEA powder (in stoichiometric ratio) have been assessed by changing the time of milling from 0 h up to 50 h while keeping the rotations per minute constant (250 RPM) under an argon atmosphere.

The XRD results show that after only 1 h of milling, the powders formed four phases: a main BCC1 phase and secondary BCC2, HCP1, and HCP2 phases. The powder microstructures are also significantly altered, as evidenced by SEM and EDS images, even though the segregation of all elements is very pronounced. After milling for 1 h, the Al element was fully dissolved into the other powders, due to higher ductility of its FCC crystal structure compared to the rest of the elements.

By increasing the milling time from 1 to 5 h, the total amount of the secondary phases decreases significantly, thus increasing the main BCC1 fraction. The particles become increasingly more homogeneous with longer mechanical alloying. However, elemental segregation is still evident. Similar particles sizes are observed up to 5 h even though the feedstock powders possessed very different morphologies before milling.

After 10 h of milling, it is possible to achieve powders comprising 95 wt.% of the BCC phase. The additional 5% relates to the HCP phases. The particle size is rapidly decreased at 10 h of milling time. This is due to the achievement of a critical value of plasticity in which the powder particles begin to fracture, thus generating new surfaces for cold welding [22].

From 20 h onwards, the powders are comprised of the pure BCC phase, acquire a globular morphology and show a significant decrease in size induced by the mechanical alloying process. However, it was previously shown that such long times of mechanical alloying may cause contamination of the powders with C and O [24,45]. Therefore, one must compromise and select parameters that avoid unnecessarily long milling times while achieving an appropriate microstructure.

The powders after 40 h of milling did not show any significant changes in comparison to those of 20 h and 30 h, as the powder morphologies are comparable and the sizes are only slightly reduced. Few elongated particles are visible after 50 h of milling, but the powders are mostly of a globular morphology.

Subsequent milling from 1 h up to 50 h induces an important broadening of the XRD peaks, as the consequence of refining the powder crystallite size by increasing plastic strains caused by repeated cold deformation from milling.

As the milling time increases (milling intensity), the lattice strain inside the powders increases due to the accumulated plastic strain caused by intensive mechanical impacts of the milling balls on the powders [22]. This is observable from the visible broadening of all lattice peaks with increasing the milling time.

From these results, it is possible to conclude that 10 h of milling seems to provide sufficiently homogeneous particles comprising an almost single-phase BCC structure with satisfactory powder size and morphologies and no significant segregation. At the same time, possible powder contamination induced by unnecessarily longer mechanical alloying can be avoided by choosing 10 h of milling.

### 4.2. Characterization of the RHEA Bulks

The produced RHEA bulks were characterized in terms of its microstructures and basic mechanical properties. Additionally, the influence of the changes in milling times on the properties of the sintered Al_0.3_NbTa_0.8_Ti_1.5_V_0.2_Zr RHEA bulks (in stoichiometric ratio) by SPS was evaluated. The precursor powders were submitted to various milling times, and powders from the selected milling times of 3, 10, 30, and 50 h were sintered by SPS.

The resulting sintered bulks appear to be 100% crystalline, according to the XRD and EBSD results, as no amorphous area was detected. The BCC phase is the predominant structure present in all sintered bulks, with similar lattice parameters as those shown by the main BCC in the powders. Despite the HCP phase being formed on the powders during milling, this phase was not exhibited in the sintered alloys. The HCP_A3 phase was predicted by CALPHAD calculations at temperatures below 600 °C. The absence of HCP on the bulk RHEAs may be explained by the relatively fast cooling after sintering, which in turn prevented its formation.

A second BCC2 phase was detected for the bulks that were produced by feedstock powders milled for both 10 and 30 h. Two BCC phases were proven to exist in similar alloys in a BCC_A2/BCC_B2 relationship, as a result of spinodal decomposition [15,16,17]. In fact, other studies [12] have shown lattice parameters in their coherent BCC_A2/BCC_B2, exhibiting values of a_BCC_A2_ = 3.3027 Å/a_BCC_B2_ = 3.3269 Å upon certain thermal treatments when one produces this alloy by arc melting, exhibiting a nanometer scale distribution of these phases. These lattices are very similar to the ones obtained in the present study for both BCC1 and BCC2 phases, indicating that they might have a BCC_A2 and BCC_B2 relationship. Both BCC phases were predicted by the CALPHAD method using the ThermoCalc software as well, in agreement with the experimental results shown here.

Additional studies [46] regarding the stability of the BCC_B2 phase in ternary Ti-25Al-25Zr alloys show that the B2 phase may transform into Ti_2_AlZr (P63/mmc, D_019_) and Al_3_Zr_5_ (P63/mcm), and an ordered orthorhombic phase during aging and mechanical polishing. None of these phases were found in our alloys, and neither were they predicted by CALPHAD.

CALPHAD calculations predicted the formation of ordered AlZr_2__B8_2_ at temperatures below 950 °C. This phase was reported in a similar RHEA [47], where the authors suggest that the B8_2_ phase is generated from the B2 phase. This phase, however, was not detected in our alloys, since the sintering took place at 1200 °C and due to the relatively fast cooling during sintering combined with a selected short sintering time.

Before sintering, the powders did not present peaks corresponding to the ZrC structure, as opposed to the sintered alloys where ZrC appeared in all cases. Additionally, increased milling times of the powders lead to the formation of a higher fraction of ZrC after sintering, indicating that contamination might be associated with prolonged milling times. For instance, the powders milled for 3 h showed less ZrC formation (2.0 wt.%) after sintering than powders milled for 50 h (6.8 wt.% after sintering), while all the sintering conditions were guaranteed to be the same. This is in agreement with other studies [24]. The sintering seems to promote the formation of the cubic structure Fm-3m of ZrC and possibly contributes to further contamination due to the use of graphite dies, even though a protective NB coating was used on the die walls. The graphite dies have already been reported to play an important role in the contamination of the sintered bulks by SPS [48].

The presence of carbides and oxides, however, has been proven to contribute to the strength, high-temperature stability, and wear resistance of metallic materials [26,27,49], including HEAs [20,21,29,50,51,52], similar to the strengthening effect used in advanced oxide dispersion strengthened (ODS) superalloys [25]. Often, the strengthening effect due to the formation of oxides and carbides is followed by a decrease in ductility [53].

It is also possible that more carbon was introduced to the powder during milling, due to the wear of the milling jar and balls. This is possibly caused by the high carbon content of the milling jar (1.5 wt.%) and milling balls (1 wt.%). While it simply precipitates as carbides during sintering, more milling appears to lead to more carbide formation. It seems that the formation of zirconium carbide is the most thermodynamically favorable in the given system and used conditions, pertaining to its highest stability, with respect to other possible carbides of other elements.

In particular, the presence of carbides and oxides in materials produced by powder metallurgy may avoid excessive grain growth by grain boundary pinning caused by the heavily plastically deformed powders due to the milling. The rapid densification might also play a role in this phenomenon [20].

In the cases of the materials produced using powders milled for 30 and 50 h, additional formation of the TiO_2_ crystal structure, belonging to the tetragonal crystal system, was observed after sintering. This structure was not observed in the respective powder forms, thus indicating that the sintering process also plays an important role in the formation of oxides.

The BCC1 predominant phase has comparable chemical compositions for all bulks. Nevertheless, the occurrence of Fe is revealed after 30 h of milling at 1.8 at.%. The presence of Fe increases for 50 h of milling up to 2.2 at.%. This contamination is also caused during the mechanical alloying process due to the wear of the equipment surfaces, namely of the milling balls and jar after long milling times.

Additionally, the increase in milling time is directly proportional to the increase in microstructural refinement. This is because the powders’ particles sizes were reduced for longer milling times, therefore forming microstructures with lower average grain sizes. This is evident by comparing the grain sizes of the bulk matrices, produced from powders milled for 3 h (33.8 µm) and 50 h (25.2 µm). Other studies [22,54] already proposed that the behavior of the powders in terms of rate refinement of its structure can be considered logarithmic with the milling time.

One of the main advantages of materials produced by powder metallurgy, as compared to the liquid phase route, is a partial or complete lack of texture [22]. Therefore, the fine powders are sintered together at high temperatures, resulting in a very homogenous microstructure, which effectively removed all the possible texture that could be present in the feedstock materials. As the bulk materials are not further processed by other forming methods (such as uniaxial hot or cold-rolling), the formation of preferential texture is prevented.

In summary, it is possible to suggest that for the case of Al_0.3_NbTa_0.8_Ti_1.5_V_0.2_Zr RHEA sintered bulks, the longer the milling time, the more refined the microstructure comprising a higher content of carbides/oxides dispersed into the BCC matrix will be.

### 4.3. Mechanical Properties

The milling time also seems to play an important role in the mechanical properties of the produced sintered bulks, as it alters the final microstructure of the alloys. This is explained by the fact that longer mechanical alloying of powders generates more severely deformed powders, thus reducing the powder size and altering their morphologies, consequently creating more refined microstructures. The penetration depth of the nanoindentation testing decreases with an increasing milling time. This is a direct consequence of the severe plastic deformation caused by the prolonged mechanical milling, which results in a rise in hardness proportionally with the time of milling.

Therefore, it was possible to see that the sintered bulks produced using 3 h of milling of feedstock powders exhibit the lowest hardness and elastic modulus among all alloys, while an increase in the milling time generates sintered bulks with increasingly higher hardness and elastic moduli. The sintered alloy corresponding to 50 h of mechanical alloying exhibits approximately 14% higher hardness than the alloy produced with 3 h of milling, while the elastic modulus of the sintered bulks increased by 3% with increasing milling times.

The hardness of the bulk RHEAs studied herein exhibit values superior to many other RHEAs reported in the literature, as shown in the comparisons in Table 6.

This is especially attributed to the choice of the powder metallurgy production route, as compared to liquid routes such as arc-melting, which show significantly lower values for similar refractory HEAs. When the powder metallurgy route is chosen, increased milling times combined with short sintering times lead to more fine-grained microstructures compared to cast materials, which can importantly contribute to the enhancement of the mechanical properties, such as hardness and strength [20], due to the possible Hall–Petch strengthening effect. Moreover, the addition of hard elements may significantly contribute to the overall hardness of the materials. For instance, one can see that the PM Al_0.3_NbTa_0.8_Ti_1.5_V_0.2_Zr alloys in this study showed values of 676 HV, while a NbTaTiV cast alloy was reported to have values as low as 298 HV.

Furthermore, pure refractories, such as tungsten (350 HV [21]), possess considerably lower hardness values than RHEAs, while displaying a much higher density of 19.25 g/cm³ [55] at 293 K in comparison to Al_0.3_NbTa_0.8_Ti_1.5_V_0.2_Zr, which has values of about 7.64 g/cm³ [12].

## 5. Conclusions

In this work, the refractory high-entropy alloy Al_0.3_NbTa_0.8_Ti_1.5_V_0.2_Zr was prepared by powder metallurgy by means of mechanical alloying and subsequent solid state sintering. The powder milling times were varied to optimize the final microstructure of the bulks.

- Longer milling times used for powder preparation for mechanical alloying produce more refined microstructures in the bulks.
- Powder homogeneity increases with the milling time, while a sufficient state is achieved after 10h of milling.
- Longer milling times lead to an increase in the total amount of oxides, carbides dispersed and iron in the matrix, compared to the non-milled state due to contamination.
- Carbide formation in the sintered RHEAs was observed after 3 h of powder milling, while oxides were detected after 30 h despite the fact that the fabrication was entirely performed using protective atmospheres. These could potentially positively contribute to the strength of the material.
- The extent of contamination is a function of the powder milling time, positively increasing with increasing milling time.
- Milling for 10 h provides the best balance between sufficient microstructural refinement, homogeneity, high hardness and minimal contamination.

## Figures and Tables

**Table 1:** Lattice parameters of the mechanically alloyed powders detected by XRD analysis.

	1 h			3 h			5 h		
	a [Å]	c [Å]	wt.%	a [Å]	c [Å]	wt.%	a [Å]	c [Å]	wt.%
BCC1	3.30	-	56.2	3.31	-	58.6	3.31	-	87.7
BCC2	3.03	-	2.9	3.03	-	3.3	3.03	-	0.4
HCP1	3.23	5.14	23.1	3.24	5.14	20.7	3.24	5.13	10.8
HCP2	2.95	4.68	17.8	2.95	4.68	17.4	2.94	4.68	1.1
	**10 h**			**20 h**			**30 h**		
BCC1	3.31	-	94.3	3.31	-	100.0	3.32	-	100.0
HCP1	3.24	5.13	4.9	-	-	-	-	-	-
HCP2	2.95	4.68	0.8	-	-	-	-	-	-
	**40 h**			**50 h**					
BCC1	3.32	-	100.0	3.33	-	100.0			

**Table 2:** Lattice parameters of the sintered bulks utilizing powders with different times of milling.

	BCC1		BCC2		ZrC (Fm-3m)		TiO_2_ (I41/amd)		
	a	wt.%	A	wt.%	a	wt.%	a	c	wt.%
Bulk 3 h	3.32	98.0	-	-	4.62	2.0	-	-	-
Bulk 10 h	3.32	52.7	3.30	43.3	4.62	4.0	-	-	-
Bulk 30 h	3.32	74.6	3.29	20.7	4.61	4.3	3.80	9.56	0.4
Bulk 50 h	3.31	88.7	-	-	4.60	6.8	3.79	9.55	4.5

**Table 3:** Chemical compositions in at.% of the BCC matrix of sintered bulks subject to different milling times performed by SEM/EDS.

(at.%)	Bulk 3 h (BCC1)	Bulk 10 h (BCC1)	Bulk 30 h (BCC1)	Bulk 50 h (BCC1)
Al	5.4 ± 0.2	6.1 ± 0.1	6.0 ± 0.2	6.1 ± 0.1
Ti	29.4 ± 1.2	31.0 ± 0.2	30.3 ± 0.1	29.8 ± 0.3
V	3.9 ± 0.6	4.0 ± 0.4	4.5 ± 0.1	4.8 ± 0.1
Zr	18.9 ± 1.2	20.5 ± 0.3	19.3 ± 0.3	20.5 ± 0.1
Nb	23.0 ± 0.8	20.2 ± 0.1	19.9 ± 0.1	19.7 ± 0.3
Ta	19.4 ± 1.2	18.3 ± 0.1	18.3 ± 0.1	18.0 ± 0.3
Fe	-	-	1.8 ± 0.1	2.2 ± 0.1

**Table 4:** Hardness and elastic modulus of the Al_0.3_NbTa_0.8_Ti_1.5_V_0.2_Zr RHEA bulks produced in this study. NHT stands for nanoindentation hardness test.

Sample	Hardness (HV 0.2)	Elastic Modulus (NHT) (GPa)	Penetration Depth (NHT) (nm)
Bulk 3 h	592.9 ± 21.0	134.7 ± 1.0	911.9 ± 5.2
Bulk 10 h	641.8 ± 9.4	136.6 ± 1.2	861.3 ± 4.9
Bulk 30 h	670.3 ± 12.3	137.2 ± 0.7	843.4 ± 6.3
Bulk 50 h	675.7 ± 9.8	138.7 ± 0.8	842.6 ± 3.6

**Table 5:** Mechanical properties of the bulk Al_0.3_NbTa_0.8_Ti_1.5_V_0.2_Zr RHEA using feedstock powders milled for 10 h. NHT stands for nanoindentation hardness test, while US means ultrasound method.

Elastic Modulus (NHT) (GPa)	Elastic Modulus (US) (GPa)	Poisson Ratio (US) [-]	Shear Modulus (US) (GPa)
136.6	136.0	0.326	53

**Table 6:** Hardness values of several HEAs found in the literature including the method of preparation, in comparison to the alloys produced in this work. The total milling times were discriminated, as well as the SPS conditions of maximum temperature of sintering and dwell time at this temperature for the referenced alloys.

Composition	Vickers Hardness (HV)	Preparation	Reference
**Al_0.3_NbTa_0.8_Ti_1.5_V_0.2_Zr**	**676**	**PM—as-sintered: Milling 50 h** **SPS: 1200 °C—15 min**	**This work**
W_0.7_(TaTiCrV)_0.3_	671	PM—as-sintered: Milling 3 h SPS: 1600 °C—10 min	[21]
**Al_0.3_NbTa_0.8_Ti_1.5_V_0.2_Zr**	**670**	**PM—as-sintered: Milling 30 h** **SPS: 1200 °C—15 min**	**This work**
**Al_0.3_NbTa_0.8_Ti_1.5_V_0.2_Zr**	**642**	**PM—as-sintered: Milling 10 h** **SPS: 1200 °C—15 min**	**This work**
FeNiCrCo_0.3_Al_0.7_	624	PM: as-sintered: Milling 45 h SPS: 1000 °C—8 min	[36]
**Al_0.3_NbTa_0.8_Ti_1.5_V_0.2_Zr**	**593**	**PM—as-sintered: Milling 3 h** **SPS: 1200 °C—15 min**	**This work**
HfTaTiNbZr	592	PM—as-sintered: Milling 1 h SPS: 1300 °C—10 min	[1,37]
Al_0.75_FeNiCrCo	577	PM—as-sintered: Milling 45 h SPS: 1000 °C—8 min	[38]
HfNbTaTiVZr	558	As-cast (Arc melting)	[1,39]
MoNbTaVW	535	As-cast (Arc melting)	[40]
W_0.9_(TaTiCrV)_0.1_	480	PM—as-sintered: Milling 3 h SPS: 1600 °C—10 min	[21]
NbTaTiVW	447	As-cast (Arc melting)	[41]
Al_0.6_NiFeCr	431	PM—as-sintered: Milling 38 h SPS: 1000 °C—1 h	[42]
HfNbTaTiZr	390	As-cast (Arc melting)	[43]
NbTiVZr	335	Annealed (Arc melting)	[1,44]
NbTaTiV	298	As-cast (Arc melting)	[41]

## Data Availability

The data presented in this study are available on request from the corresponding author.
